# Anxiety, Coping, and Self-Efficacy as a Psychological Adjustment in Mothers Who Have Experienced a Preterm Birth

**DOI:** 10.3390/jcm14124174

**Published:** 2025-06-12

**Authors:** Agata Białas, Karolina Kamecka, Paweł Rasmus, Dariusz Timler, Remigiusz Kozłowski, Anna Lipert

**Affiliations:** 1 Department of Preventive Medicine, Medical University of Lodz, 90-752 Lodz, Poland; agawhite@znajomi.pl; 2 Department of Management and Logistics in Healthcare, Medical University of Lodz, 90-419 Lodz, Poland; kkamecka@gmail.com (K.K.); remigiusz.kozlowski@umed.lodz.pl (R.K.); 3 Department of Medical Psychology, Medical University of Lodz, 90-131 Lodz, Poland; pawel.rasmus@umed.lodz.pl; 4 Department of Emergency Medicine and Disaster Medicine, Medical University of Lodz, 90-419 Lodz, Poland; dariusz.timler@umed.lodz.pl

**Keywords:** postpartum period, mother–child relations, stress, anxiety, emotional support program

## Abstract

**Background/Objectives:** Research shows that mothers of premature infants can experience increased symptoms of anxiety, depression, and even a post-traumatic stress in comparison to mothers of healthy, full-term infants. The aim of this study was to analyze and compare anxiety, coping, and self-efficacy in mothers who have and have not experienced a preterm birth, providing a basis for developing a targeted, mother-oriented support program that supports their adjustment to difficult situations. **Methods:** The study included 251 women, 112 of whom delivered infants prematurely (PTB group) and 139 who delivered infants at term (T-B group). Data were collected by using (1) The State-Trait Anxiety Inventory (STAI) Questionnaire, (2) the Generalized Self-Efficacy Scale (GSES), and (3) the Coping Inventory for Stressful Situations Questionnaire (CISS). **Results:** PTB women had higher results in anxiety in comparison to T-B women. Also, they were characterized by statistically significantly lower generalized self-efficacy and ability to cope with stress. PTB women more often presented the emotion-oriented coping style. **Conclusions:** A mother-oriented support program based on personal resources is a solution which could help mothers better adjust to difficult situations related to preterm birth child treatment and care.

## 1. Introduction

Approximately 15 million babies are born preterm annually worldwide [1]. It was estimated that the global preterm birth rate in 2014 reached 10.6% of live births [2]. Analyzing the phenomenon regionally, the preterm birth rates for 2014 ranged from 8.7% in Europe to 13.4% in North Africa, and this trend is increasing, especially in high-income and high–middle-income countries [2,3]. Low- and middle-income countries report approx. 3.8 million premature births annually. Southeast Asia and sub-Saharan Africa record nearly two thirds of the total of prematurely born infants and over three quarters of the global number of infant deaths caused by premature birth and postnatal complications [4]. In recent decades, the overall rate of premature births has also increased in European countries, including Poland [5]. There are variations in newborn children’s chances of survival, which depend on their place of birth. The advancement in neonatal intensive care in the last quarter of the 20th century has increased the chance of potential survival at a lower gestational age for 1.2 million newborns [5]. The regionalization of obstetric and neonatal care and the transfer of pregnant women at risk of premature birth to a center with a third level of reference allowed for a certain reduction in mortality and morbidity among premature infants [6]. Also, ongoing improvement in technology in neonatal intensive therapy can help to lower the limits of birth weight and gestational age for survival [7].

Preterm birth is defined as a birth occurring prior to 37 weeks of gestation, whereas infants born before 28 weeks of gestation are referred to as extremely preterm [8]. Preterm newborns are typically characterized by immature or extremely immature systems and organs. The immaturity of organs in preterm and extremely preterm infants classifies their condition as serious [9,10]. Recently, the ELGAN study of the brain and related disorders in extremely low-gestational-age newborns provided evidence that brain damage is associated with microorganisms in placenta parenchyma [11]. That is, however, only a part of the complex issue of premature birth and infant conditions, because a significant component also involves the impact of preterm delivery on the mental condition of their mothers [12,13]. These are not specific diagnosed mental health problems in the form of some disease entity, but rather, emotional ones that, in the long term, may lead to the development of more serious mental disorders. Research focusing on psychological distress in the weeks after birth and the first months of life consistently shows that parents of premature infants experience increased symptoms of depression, anxiety, and post-traumatic stress compared with new parents of healthy, full-term infants [14]. There is also cross-sectional evidence of increased psychological distress among mothers of preschool children born prematurely [14]. Also, stress is increased by the infant’s hospitalization in neonatal intensive care units (NICUs), where they undergo intensive medical treatment. Mothers often feel helpless and have no sense of control over the situation they are experiencing. This results from separation from their babies, and mothers can struggle with a range of negative emotions such as shock, anxiety, anger, and guilt [15]. According to research conducted on parents whose children were treated in neonatal care units in 2007–2008 in Poznan, anxiety was a prevailing emotion experienced by mothers [16]. More importantly, the consequences of preterm delivery may not subside for many years to come. Nearly 50% of mothers suffer from post-traumatic stress disorder (PTSD), which might affect their motherhood [16]. Moreover, it was found that the cry of premature infants, which contains information about their current level of suffering, as well as general fitness, is perceived by mothers as more aversive and physiologically arousing. As a result, all of these infant characteristics and signals may complicate parental bonding because mothers may be hesitant to bond with a premature infant with poor survival prospects and possible developmental difficulties [17]. Consequently, the emotional needs of the mothers after preterm labor should be a vital element of care provision, as the preterm infants’ psychomotor development relies heavily on their mother’s emotional status [15,18]. Also, it is crucial to support psychological adjustment, which refers to psychological processes in response to difficult health conditions or situations associated with treatment. That response can be positive or negative, depending on the person’s individual resources, enabling an appropriate response [19].

Although a lot of research on premature babies can be found in the scientific literature, there are few studies focusing on parents of premature babies and almost none conducted on the Polish population [20,21]. One study conducted in 2014 showed that preterm birth mothers presented major psychological distress; however, the results were obtained using a small and rather specific socio-demographic group of women, which did not allow for a full generalization of the results [22]. Data from a more recent study (2023) among preterm birth mothers examined only anxiety in relation to depression but did not include an assessment of the individual resources that are important for psychological adjustment to such a difficult situation [23]. The findings from Kozel et. al. examined the relationship between optimism and social support and emotion management; however, the sample included women at risk of preterm birth, so before the traumatic event had happened [24]. Several studies have focused on the psychological effects of time spent at the NICU, but the samples included participants of strong disparities, e.g., non-coparenting parents and parents interviewed when their children were between 15 months and 8 years of age [25]. To our knowledge, only one study has been conducted in Poland so far on parents of prematurely born children, in which attention was drawn to the aspect of providing support to mothers of premature babies; the lack of access to a such support was emphasized [26]. However, little has been changed since that time.

Although psychological screening and supportive interventions are recommended for parents of high-risk infants [27], there are still only sporadic psychiatric or psychological consultations for pre-term birth mothers, which are often not refundable by health insurance providers. Therefore, the aim of this study was to analyze and compare anxiety, coping, and self-efficacy in mothers who have and have not experienced a preterm birth. The findings will expand current knowledge, filling the existing gap in psychological screening data among pre-term birth mothers. Also, the findings may provide the basis for developing a specially tailored support program helping achieve better adjustment to the difficult situation related to preterm birth child treatment and care. We hypothesize that pre-term birth mothers, who are at higher risk of anxiety in comparison to term birth mothers, can be characterized as having weak abilities of self-efficacy and coping. Also, due to the situation of premature birth, these women may use other types of coping strategies what should be taken into account when developing psychosocial intervention programs to help pre-term birth mothers adjust to parenthood.

## 2. Materials and Methods

### 2.1. Procedure

**Figure 1:**
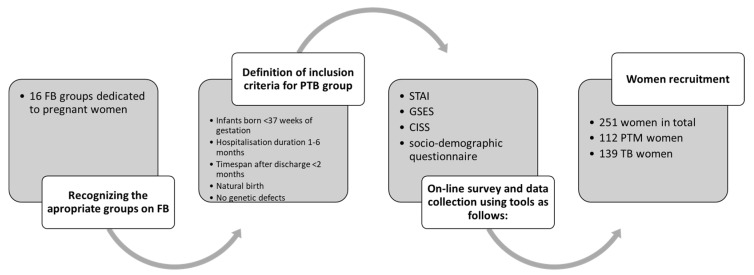
Data collection protocol.

Two hundred and fifty-one women were included in the study, 112 of whom delivered infants prematurely and 139 who delivered infants at term. The newborns were hospitalized in 17 hospitals throughout Poland. The study group was divided into preterm birth mothers (PTB) and term birth mothers (T-B). Both groups had to be characterized by relatively similar socio-demographic characteristics, and the only difference was that the PTB group included mothers who gave birth to children prematurely, i.e., infants were born before 37 weeks of gestation. The sample was determined using a purposive sampling technique which allowed us to collect data focusing on specific people with rare experiences; the findings application was addressed to a specific population. Figure 1 presents the data collection protocol. The following inclusion criteria were applied for the PTB group: (1) infants born before 37 weeks of gestation; (2) duration of the infant hospitalization not shorter than one month and no longer than 6 months; (3) timespan after discharge from hospital of infant not longer than 2 months; (4) natural birth—a birth that did not end in a cesarean; and (5) no genetic defects in preterm infants. The T-B group (C) was composed of women included based on the following inclusion criteria: (1) infants born after 37 weeks of gestation; (2) duration of hospital stay not longer than 3 days; (3) timespan after discharge from hospital not longer than 2 months; (4) natural birth; and (5) no genetic defects in newborns.

### 2.2. Methods

The study was conducted in the period from 15 January 2021 to 15 June 2022 using the following research tools: (1) the State-Trait Anxiety Inventory (STAI) Questionnaire, (2) the Generalized Self-Efficacy Scale (GSES), (3) the Coping Inventory for Stressful Situations Questionnaire (CISS), and (4) a specially designed questionnaire to obtain the characteristics of the study population of preterm infants’ mothers and socio-demographic data. After analysis of the available statistical data related to preterm birth events during the year, it was estimated how many preterm birth events could occur during the year and therefore how many mothers could be enrolled. A sample size of around 100 women was determined to be enough based on previous publications which described data collected from a smaller group (experience-based sample size assessment). Also, assuming a response rate of 5%, it was estimated how much time it would take to collect responses from the appropriate number of women.

The survey questionnaires were available on-line and were distributed to 16 groups on Facebook dedicated to mothers of premature babies and 5 groups dedicated to mothers of newborn babies. Due to the COVID-19 pandemic, this was the only possible means of reaching the study group allowed by health care facilities at that time. The questionnaires were anonymous and their completion was indicated as being equivalent to agreeing to participate in the study. Participation in the study was voluntary and every person could opt out at any time. The research was performed in accordance with relevant guidelines, and informed consent was obtained from all the participants. This study was waived by the Bioethics Committee of Medical University of Lodz (RNN/170/21/KE), as it did not reveal the characteristics of a medical experiment or a clinical trial performed on a patient. The study was a continuation of an ongoing research to determine separate but complementary conclusions [28].

### 2.3. Tools

The STAI Questionnaire is used to diagnose both state anxiety (situation-related anxiety) and trait anxiety as a relatively permanent personality trait (anxiety as a feature) [29,30]. STAI is a commonly used measure of trait and state anxiety. It can be used in clinical settings to diagnose anxiety and to distinguish it from depressive syndromes. It is frequently used in research as an indicator of stress and routinely used to assess anxiety in standard clinical practice. The questionnaire is divided into two parts, each containing 20 questions pertaining to state anxiety (Questionnaire X1) and trait anxiety (Questionnaire X2). The scores that can be obtained for each part of STAI are in the range from 20 to 80 points. The results are interpreted by means of sten scores standardized for sex and age. Sten scores are standardized in the range of 1–10, where scores of 1–4 indicate low levels of anxiety, 5–6 indicate average levels of anxiety, and 7–10 indicate high levels of anxiety [31]. The STAI Questionnaire has been used in the other studies conducted at neonatal intensive care units [32]. The questionnaire has been validated on the Polish population, and Cronbach’s alpha for state anxiety (STAI-S) was 0.958 and for trait anxiety was (STAI-T) 0.850 [33].

The Generalized Self-Efficacy Scale (GSES) is used to obtain a general sense of perceived self-efficacy in handling difficult situations and life hurdles [29]. The sense of self-efficacy is defined as the conviction of being capable of carrying out certain activities or achieving goals. The scale consists of 10 statements which are a part of one factor; the results are calculated according to the key that should be interpreted by means of sten scores. The internal reliability of the Polish version of GSES has been reported to be good, with Cronbach alpha coefficient = 0.85 [30].

The Coping Inventory for Stressful Situations Questionnaire (CISS) consists of 48 statements describing human behavior in stressful situations [34]. Respondents self-assess their behavior using a five-point Likert scale describing the frequency of certain coping methods used in stressful situations. The CISS questionnaire distinguishes among three basic coping strategies [35]: (a) Task-Oriented (T) strategy refers to coping with stress by taking on tasks; (b) Emotion-Oriented (E) strategy applies to people who, when tackling stressful situations, tend to concentrate on themselves, their emotions such as anger, sense of guilt, and tension; (c) Avoidance (A) strategy describes the coping style typical of people who tend to reject thinking and experiencing stressful adversity. The avoidance-oriented style may take two forms: Distraction (A-D) i.e., substituting an alternative task, or Social Diversion (A-SD) i.e., seeking social contact [36]. In CISS, the score is calculated on three 16-item scales: T, E, and A. The total points in each scale is the raw result. The obtained raw results should then be referred to sten scores from the relevant table in an annex [37]. The reliability of the Polish version of the CISS questionnaire, measured with the Cronbach’s alpha coefficient, ranges from 0.72 to 0.92 [38,39].

### 2.4. Ethics

This study was waived by the Bioethics Committee of Medical University of Lodz (RNN/170/21/KE), as it does not reveal the characteristics of a medical experiment or a clinical trial performed on a patient.

All the psychological tests and questionnaires used for the study are copyrighted. All the necessary permissions/licenses were obtained for the administration of questionnaires. The administration of the aforementioned questionnaires was possible because a co-author and one of the researchers in the study is a qualified psychologist with specialization in clinical psychology, so he has a full right to use these types of diagnostic tools. Moreover, the institution with which the psychologist is affiliated has a permanent paid license to use the tools applied in the study. The additional, specially designed questionnaire to collect socio-demographic data was pre-tested before being used in the main phase of the study. Data on the newborn’s Apgar scale score were collected by asking about it in the questionnaire. Mothers were asked to answer according to the medical records of their children.

### 2.5. Statistical Analysis

Statistical analyses were performed using Statistical version 13.1 software (StatSoft, Tulsa, OK, USA). Referring to previous population-based studies [40,41], the descriptive statistics were presented as percentages for dichotomous variables, and chi^2^ test was used for the comparison of percentages. Continuous variables are presented as median and confidence intervals. The effect size was determined by Cohen’s d value, defined as the difference between two means divided by a standard deviation for the data. Effect sizes were recorded as small (d = 0.20–0.49), medium (d = 0.51–0.79), and large (d ≥ 0.80). The data were not normally distributed (Shapiro–Wilk test for normal distribution analysis), so the Mann–Whitney test was used to analyze the differences between the groups. Significant differences were accepted for all analyses at the level of *p* < 0.05.

## 3. Results

### 3.1. Characteristics of the Study Group

The study involved 251 women who were divided into two groups: the preterm birth mothers group (PTB) that consisted of 112 women, and the term birth mothers group (T-B) including 139 women. Women were divided according to whether they had delivered at term or prematurely. In PTB, the infants obtained 7 points in the Apgar scale in comparison to T-B, where 10 points were given to each newborn (Table 1). Most of the women in both PTB and T-B were married and had higher education, and almost half of them came from a city with a population over 50,000 citizens. The average work experience was 11 years for PTB and 13 years for T-B. No differences were observed in the self-assessment of physical and mental health, which both groups assessed as good (Table 1).

### 3.2. The Analysis of the Questionnaire Data

The study women declared to have been supported by their families during pregnancy and at childbirth. Over a half of PTB reported considerations concerning potential premature termination of their pregnancy. Although both PTB and T-B reported that the information about their infants they received from the medical staff was sufficient, most of the women reported that no psychiatric consultation was available/provided at the hospital. Only 15% of PTB declared to have received some psychological consultation at the hospital (Table 2).

### 3.3. Psychological Analysis

Generally, the women from PTB were characterized by lower results obtained in psychological tests assessing generalized self-efficacy in the GSES test and the ability to cope with stress in the CISS test than the women from FTB (Table 3). A statistically significant difference and small size effect was observed in the GSES results with *p* < 0.05. Also, the PTB women obtained lower scores in the CISS test, irrespective of their coping strategy. No statistically significant difference but a small size effect was observed in women presenting the task-oriented coping style. In addition, a significant difference and large size effect was observed in the results of women presenting the emotion-oriented coping style, with *p* < 0.0001 (Table 3). There was no statistically significant differences and no effect size in the results of the CISS test related to the avoidance-oriented style (Table 3). Moreover, PTB had higher results in the assessment of anxiety (STAI), but the difference was not statistically significant (Table 3).

## 4. Discussion

The post-preterm delivery circumstances and preterm infant stay at neonatal intensive care units are regarded as early adverse experiences which may affect mothers’ emotional state and, consequently, lead to difficulties in establishing relationships and bonds between the mother and the newborn [37]. The findings of the present study showed that the adversity of preterm delivery results in a reduction of the sense of self-efficacy in preterm birth mothers, and that their coping strategies are more concentrated on experiencing emotions related to the consequences of preterm delivery. It addition, anxiety was observed to be greater in PTB women, although it was not much greater compared to women who had delivery at term. These results fills a gap in current knowledge, providing data for more effective management of and support for preterm infants’ mothers in their hardship. Good mental health and a stable emotional state of the mother are positively crucial values in the care given to the newborn. Multiple studies have emphasized the relationship between psychomotor, socioemotional, and cognitive development in infants and their mothers’ emotional state [42].

In general, in our study, the preterm infants’ mothers did not differ in terms of their age or education. In the presents study, the PTB mothers felt greater anxiety and stress in comparison to the T-B mothers. This may have resulted from the limited number of mothers who declared to receive psychological support from hospital. With regard to psychiatric consultations, only two mothers benefited from the provision of such assistance. Perinatal depression and anxiety are common worldwide, but in preterm infants’ mothers, depression, anxiety, and post-traumatic stress disorder are recorded with increased frequency [43]. The frequency of post-preterm delivery stress can be estimated at even 70% [37]. A substantial majority of preterm infants received an Apgar score of 7 at 5 min, which, according with the Neonatal Encephalopathy and Neurologic Outcome, is defined as reassuring [44]. That score could have influenced the sense of anxiety of PTB mothers, because the score did not correspond to the long-term prognosis of preterm infants. Although the Apgar score is still commonly used in preterm infants as a useful tool in short-term prognosis in preterm infants, its usefulness is controversial with regard to morbidity in the newborn period.

The women from the PTB group were characterized with worse self-efficacy and the ability to cope with stress than the women from T-B group. According to self-efficacy theory, a higher level of GSE helps people to perceive potentially difficult situations as being more manageable which, in turn, can make the person feel less stressed [45]. A study on pregnant women facing possible preterm labor evidenced that increased self-efficacy can be triggered by dispositional optimism [46].

In our study, the analyzed women differed also in the result of the coping level and type of coping strategies. PTB women obtained lower scores in the CISS test and were characterized by the emotion-oriented coping style, which is a consequence of the feelings experienced in the adversity of preterm delivery and the unstable condition of the infant. People with emotion-oriented coping styles are more effective in the reduction of emotional distress related to difficult situations, rather than changing the actual situation [47]. A recent study evidenced that applying problem-solving skills empowered mothers in NICU and increased their emotional self-efficacy [48].

Elevated anxiety levels and depression symptoms, along with an increase in negative experience, helplessness, exclusion, and alienation, may enhance the suffering and despair and adversely affect positive parenting [49]. Therefore, it is important that tailored interventions for better adjustment to the difficult situation faced by PTB mothers should be complex, consolidating knowledge from different specialists. Healthcare professionals should consider, e.g., the complex role of self-efficacy, and understand how individual differences in GSE may influence stress perception [49]. Social support is of key importance in health and well-being; a lack of such support makes proper rest impossible and may even result in self-care deficits. What is available to at term neonates’ mothers is severely limited for the mothers of preterm infants [43].

So far, the developed intervention programs has focused only on supporting premature babies to compensate for neurodevelopmental deficits. No supportive programs of mental health have been prepared for parents who are burdened with the situation of a preterm birth, e.g., the responsibility and constant threat resulting from the health condition of a prematurely born child [50,51]. Although Family-Centered Care (FCC) has become the gold standard of intensive care unit practice, the implementation of FCC in clinical practice remains inconsistent [52]. Due to this fact, there is no significant evidence regarding the effectiveness of selected parent support programs and interventions in NICU [53]. Despite general improvements in care for premature newborns, the findings of the present study indicate that PTB mothers still feel great anxiety, which can significantly affect their parenting skills. In addition, PTB women are characterized by a specific, emotional-oriented coping strategy. However, there is still a lack of sufficient psychological support, i.e., provided by general health insurers, for PTB mothers, who generally have reduced coping skills. It appears obvious that professional mother-oriented support after preterm delivery and training for medical personnel concerning communication with patients and their families are necessary [54,55]. However, these tasks are quite demanding, as passing on information is one of the most arduous tasks for medical personnel, and medical educations do not include formal training in that respect. Moreover, communication skills training is not included in medical faculties’ curriculum in many countries. Therefore, the training given to medical personnel on communication with PTB mothers should be an intrinsic element of the skills improvement process [54,55].

There is a need to create a special mother-oriented support program based on the individual diagnosis of the mother’s condition and resources during their stay in the hospital and after hospitalization. Holistic care for PTB mothers requires the development and implementation of educational programs including psychological care focused on their individual needs and psychological adjustment. Efforts should be made to optimize educational activities in the group of PTB mothers by organizing training during the hospitalization of the mother and child. Emotion-focused therapy (EFT), i.e., healing from the belief that emotions are strongly linked to identity, may be particularly important, as indicated by the results of the present study. There are two foundational skills that are important for EFT therapy outcomes: (a) mothers can manage at their emotions by increasing their awareness and acceptance of their feelings; (b) mothers can translate their emotions into helpful information as a way of preventing negative outcomes. It would also be optimal if specialists such as psychologists, psychiatrists, and psychotherapists were available at relevant hospitals to begin immediate support for mothers. The importance of mother-oriented support for mothers of premature babies should be emphasized in specially developed health campaigns to disseminate knowledge about premature babies and complications resulting from premature birth, as well as the experiences and emotions of parents of premature babies. All such activities should be reported to ministerial-level decision-makers and financed as one of the elements of primary health care.

The first strength of this study is that it is one of few in which attention is paid more to resources that allow mothers to cope in a difficult situation, rather than the mental health problems. Secondly, the data were obtained by using a standardized and validated tools used widely in many international studies [31,56,57]. Due to the fact that the data collection time coincided with the period of the COVID-19 pandemic, the survey was conducted on-line, which probably resulted in a higher responsiveness than the direct contact attempts with PTB mothers in the NICU. The on-line form of the survey gave more anonymity and encouraged mothers to be more sincere.

Some limitations should be emphasized. One limitation is related to the small sample size due to the limited number of people who responded to solicitation via Facebook. Therefore, the results should be presented with less certainty and more as a basis for future studies with larger sample sizes and better control over the information. However, the sample was still more numerous and less homogenous in comparison to previous studies conducted on PTB mothers [31,32,33]. Next, some additional issues, such as depression or PTSD prevalence could be used to help in the assessment of the mental well-being and emotions of PTB women in a more complex manner. To know the perspective of health care specialists in contact with PTB mothers, the observed problems and the relations with preterm infants’ mothers would be also beneficial. Self-report online study has its limitations, such as misunderstandings or answering the questions which in a way that gives a better picture of the person, which may introduce response biases, while the cross-sectional design limits causal inferences. Moreover, an online survey may introduce selection bias, as only mothers with internet access and familiarity with social media platforms could participate. Therefore, further studies based on additional direct tools and methods to obtain the data would be recommended.

## 5. Conclusions

Psychological adjustment in the mothers who have experienced a preterm birth is worse than that of mothers of term newborns. The adversity of preterm delivery causes that their sense of self-efficacy is reduced in comparison with the term infants’ mothers. Their coping strategies are concentrated on experiencing emotions related to the consequences of preterm delivery. Emotional and psychological support program is the solution which could undoubtedly comfort preterm infants’ mothers in their hardship.

## Figures and Tables

**Table 1:** Original survey questionnaire outcomes providing socio-demographic data, data on education, work experience, and physical and mental health; PTB group vs. T-B group.

Variables	PTB Group (N = 112)	T-B Group (N = 139)	*p* Value	d
	Median (IQR)			0.36
Age	35.0 (8.50)	37.0 (11.00)	0.008	
Work experience	11.0 (8.00)	13.0 (10.00)	0.010	0.36
Apgar score	7.0 (3.00)	10.0 (1.00)	0.0001	1.45
	N (%)			
Siblings				
Yes	53 (47.3)	67 (48.2)	0.023	-
No	59 (52.7)	72 (51.8)	0.030	-
Marital status				
Unmarried	18 (16.1)	10 (7.2)	0.066	-
Married	85 (75.9)	114 (82.0)	0.0001	-
Divorced	9 (8.0)	13 (9.3)	0.318	-
Separated	0	2 (1.4)	0.132	-
Place of residence				
City over 50,000 citizens	55 (49.1)	67 (48.2)	0.051	-
City below 50,000 citizens	25 (22.3)	31 (22.3)	0.294	-
Village	31 (27.7)	38 (27.3)	0.247	-
No answer	1 (0.9)	3 (2.1)	0.273	-
Education				
National Vocational Qualification (NVQ)	1 (0.9)	1 (0.7)	1.000	-
Secondary	19 (16.9)	15 (10.8)	0.399	-
Higher	92 (82.1)	123 (88.5)	0.0001	-
Physical health self-assessment				
Very good	16 (14.3)	29 (20.9)	0.015	-
Good	50 (44.6)	73 (52.5)	0.0002	-
Average	40 (35.7)	29 (20.9)	0.067	-
Bad	4 (3.6)	6 (4.3)	0.474	-
Very bad	1 (0.9)	0	0.237	-
No answer	1 (0.9)	2 (1.4)	0.524	-
Mental health self-assessment				
Very good	15 (13.4)	15 (18.0)	1.000	-
Good	53 (47.3)	68 (49.0)	0.015	-
Average	31 (27.7)	28 (20 1)	0.604	-
Bad	12 (10.7)	15 (10.8)	0.491	-
Very bad	1 (0.9)	2 (1.4)	0.524	-
No answer	0	1 (0.7)	0.289	-

Statistical significance for descriptive statistics was determined using chi^2^ test when *p* < 0.05.

**Table 2:** The results of the original survey questionnaire providing data on the support the mothers received from family, neonatal care specialists, and medical personnel, time devoted to the newborns’ siblings, and medical considerations concerning premature delivery, Abgar score: PTB group vs. T-B group.

	PTB Group (N = 112)	T-B Group (N = 139)	*p* Value
	N (%)		
Family support			
Yes	34 (30.3)	48 (34.5)	0.025
Rather yes	44 (39.3)	50 (36.0)	0.344
Hard to say	16 (14.3)	22 (15.8)	0.231
Rather no	11 (0.9)	13 (9.3)	0.629
No	7 (6.2)	5 (3.6)	0.504
No answer	0	1 (0.7)	0.289
Presumptions of premature termination			
Yes	63 (56.2)	42 (30.2)	0.001
Don’t know	0	1 (0.7)	0.287
No	49 (43.7)	95 (68.3)	0.0001
No answer	0	1 (0.7)	0.287
Psychological support in the hospital			
Very often	5 (4.5)	1 (0.7)	0.055
Often	12 (10.7)	3 (2.1)	0.005
Rarely	22 (19.6)	10 (7.2)	0.009
Not at all	73 (65.2)	119 (85.6)	0.0001
No answer	0	6 (4.3)	0.008
Psychiatric consultation in the hospital			
Yes	2 (1.8)	0	0.132
No	110 (98.2)	132 (95.0)	0.597
No answer	0	7 (5.0)	0.004
Information on infant’s health status provided by medical staff			
Sufficient	68 (60.7)	91 (65.5)	0.0001
Medium	33 (29.5)	28 (20.1)	0.391
Insufficient	11 (9.8)	19 (13.7)	0.081
No answer	0	1 (0.7)	0.287
Less time for newborns to spend together with their siblings			
Yes	23 (20.5)	15 (10.8)	0.105
Probably yes	17 (15.2)	19 (13.7)	0.682
Not really	10 (8.9)	24 (17.3)	0.004
No	15 (13.4)	22 (15.8)	0.159
No answer	47 (42.0)	59 (42.4)	0.058
The newborn’s siblings felt the need to talk about their feelings and fears			
Yes	13 (11.6)	22 (15.8)	0.065
Probably yes	6 (5.3)	16 (11.5)	0.014
Not really	17 (15.2)	17 12.2)	1.000
No	14 (12.5)	16 (11.5)	0.660
No answer	62 (55.3)	68 (48.9)	0.321

Statistical significance for descriptive statistics was determined using chi^2^ test when *p* < 0.05.

**Table 3:** Results of the psychological tests: PTB group vs. T-B group.

	PTB Group (n = 112)	T-B Group (N = 139)	*p* Value	d
	Median (IQR)			
GSES	28.00 (6.00)	29.00 (5.00)	**0.010**	0.29
CISS T	57.50 (16.50)	59.00 (12.00)	0.066	0.26
CISS E	4.00 (2.00)	42.00 (21.00)	**0.0001**	3.75
CISS A	37.00 (12.00)	37.00 (12.00)	0.710	0.04
CISS A-D	16.00 (7.00)	16.00 (8.00)	0.653	0.07
CISS A-SD	14.00 (5.00)	15.00 (6.00)	0.225	0.14
STAI	91.50 (36.00)	87.00 (33.00)	0.127	0.21

Statistical significance was determined using Mann–Whitney test when *p* < 0.05.

## Data Availability

The data presented in this study are available on request from the corresponding author.
